# *Salvia spinosa* L. Protects against Diabetes-Induced Nephropathy by Attenuation of Mitochondrial Oxidative Damage in Mice

**DOI:** 10.1155/2021/4657514

**Published:** 2021-12-26

**Authors:** Milad Jeshan, Fatemeh Yousefbeyk, Hiva Rahmati, Amir Hosein Shoormeij, Mitra Rezazadeh, Ehsan Zamani

**Affiliations:** ^1^Student Research Committee, School of Pharmacy, Guilan University of Medical Sciences, Rasht, Iran; ^2^Department of Pharmacology and Toxicology, School of Pharmacy, Guilan University of Medical Sciences, Rasht, Iran; ^3^Department of Pharmacognosy, School of Pharmacy, Guilan University of Medical Sciences, Rasht, Iran; ^4^Laboratory of Pathology, Imam Khomeini Hospital, Jiroft University of Medical Sciences, Jiroft, Iran

## Abstract

Mitochondrial oxidative damage is a crucial factor in the pathogenesis of diabetic nephropathy (DN), which is among the most prevalent problems of diabetes, and there hasn't been an effective treatment for DN yet. This study planned to investigate the effects of *Salvia spinosa* L. on mitochondrial function along with its protection against streptozotocin-induced nephropathy in diabetic mice. After the injection of streptozotocin (STZ) and verification of the establishment of diabetes, mice (*n* = 30) were randomly divided into the following groups: control group, diabetic-control, *S. spinosa*-treated diabetic (50, 100, and 200 mg/kg), and metformin-treated diabetic group (500 mg/kg). After four weeks of treatment, the mice were weighed. Blood and kidney tissues were examined for biochemical and histological evaluation. Hematoxylin and eosin staining was used for evaluating renal pathologic damage. Oxidative damage in the kidney was assessed by the evaluation of lipid peroxidation and glutathione oxidation. Furthermore, differential centrifugation was used to obtain the isolated mitochondria, and mitochondrial toxicity endpoints (mitochondrial function and mitochondrial oxidative markers) were determined in them. *S. spinosa* remarkably reduced the blood urea and creatinine concentrations, and also normalized kidney weight/body weight coefficient in the diabetic mice. *S*. *spinosa* ameliorated the incidence of glomerular and tubular pathological changes in histological analyses. Moreover, the oxidative and mitochondrial damages were notably attenuated in renal tissues of *S. spinosa*-treated mice. These results indicate that the methanolic extract of *S. spinosa* modulates the nephropathy in the diabetic mice by the amelioration of oxidatively induced mitochondrial damage and provides a reliable scientific base, suggesting *S. spinosa* as a promising alternative remedy against DN.

## 1. Introduction

Diabetes mellitus (DM) leads to profound pathological complication including diabetic nephropathy (DN, a microvascular complication), in approximately 25–35% of patients with both main types of DM [1, 2]. The progression of DN has various clinical stages, from mesangial hypertrophy and glomerular hyperfiltration with microalbuminuria to macroalbuminuria and nephrotic proteinuria, followed by chronic renal disease, which finally lead to end-stage kidney disease [3].

Regarding the complex pathogenesis of diabetes, the persistent hyperglycemia leads to an excessive reactive oxygen species (ROS) generation, oxidative stress, and mitochondrial dysfunction [4, 5]. Eventually, oxidative damage occurs in the lipids, proteins, and nucleic acids [1, 6, 7]. Moreover, the ROS play a main role in the pathogenesis of many diseases, and antioxidants are responsible for keeping the normal balance [8].

In clinical practice, controlling excessive oxidative stress, hyperglycemia, dyslipidemia, and hypertension are the important strategies to improve albuminuria and ultimately treat DN [9, 10]. Nowadays, researchers' attention is drawn to natural antioxidants on account of mediocre outcome of classic medications [7, 8].

Medicinal herbs have gained significant importance in the last few decades, and available literature shows that huge numbers have demonstrated considerable antioxidant effects on diseases including DN [2, 3, 5, 10].

*Salvia spinosa* L. is a plant belonging to the *Salvia* genus, in the Lamiaceae family, which is native to Iran. According to the previous reports, *Salvia* species are rich in bioactive compounds such as phenolic compounds, terpenoids, isoprenoids, triterpenoids, and sesterterpenoids [11]. These phytochemicals make this genus an appropriate candidate for providing a natural antioxidant [11, 12], antibacterial, anti-inflammatory, anticancer, antimalarial, antidiabetic [11, 13], and anti-Alzheimer's disease agent [8, 11–14]. For example, a study showed that the aqueous extract of *Salvia miltiorrhiza* administered to type II diabetic rats significantly decreased lipid peroxidation injury [3].

As regards the mentioned properties of *Salvia* species, this traditional herb may yield potential candidates for the development of novel therapies against DN. However, till now, the protective effects of *S. spinosa* L. have not been investigated on diabetic nephropathy. Therefore, the aim of this study was to investigate the efficacy of the methanolic extract of *S. spinosa* L. as nephroprotective in diabetic male mice.

## 2. Materials and Methods

### 2.1. Preparation of Methanol Extract of *Salvia spinosa*

*Salvia spinosa* L. aerial parts were collected from Rostamabad, Guilan, north of Iran (June 2018). The plant specimen was recorded at the herbarium of the School of Pharmacy, Guilan University of Medical Sciences, Rasht, Iran (112 HGUM).

*S. spinosa* (500 g) was dried in the shade and powdered. Eventually, we extracted the powder with methanol by the percolation technique. Then, the solvent was evaporated in a rotary evaporator to obtain the extract. Finally, the extracts were refrigerated until other assays.

### 2.2. Animals

Male albino mice (*n* = 30), weighing 25–30 g, were purchased from the Guilan University of Medical Sciences' animal breeding and care center, Rasht, Iran. The animals were kept under standard conditions with a 12-hour light: 12-hour dark cycle, 45–55% humidity, at room temperature (RT), and with free access to standard water and food during the study.

All protocols were confirmed by the Ethics Committee of the Guilan University of Medical Sciences (Ethical No.: IR.GUMS.REC.1398.358).

### 2.3. Diabetes Induction and Experimental Procedure

After overnight fasting, mice were subjected to a single *i.p.* injection of fresh streptozotocin (STZ) (45 mg/kg) prepared in sodium citrate buffer (pH 4.5). After one week of STZ injection, mice with plasma glucose higher than 250 mg/dl were considered diabetic mice. Randomly, mice were allocated into six groups (*n* = 5) as follows:

  Group I: control (nondiabetic mice treated with normal saline, *i.p.*)
  Group II: diabetic mice (treated with normal saline, *i.p.*)
  Group III–V: diabetic mice treated with *S. spinosa* doses (*i.p.*, 50, 100, and 200 mg/kg.)
  Group VI: diabetic mice treated by metformin (*i.p.*, 500 mg/kg)

Mice were euthanized by the injection of ketamine (80 mg/kg) and xylazine (5 mg/kg) after experiments according to the standard protocols [15].

### 2.4. Assessment of Fasting Blood Glucose (FBG)

At the end of the experimentation, FBG was evaluated by tail vein blood sampling with a glucometer (EasyGluco, Infopia Co., Korea) [16].

### 2.5. Evaluation of Kidney/Body Weight Coefficient

Body weight of mice was evaluated after overnight fasting. Following euthanization, the kidneys were instantly and precisely weighed. The kidney/body weight coefficient was determined as follows: kidney/body weight coefficient (g/g) equals kidney weight (g) divided by body weight (g) [2].

### 2.6. Biochemical Analyses

Blood samples were gained from retro-orbital venous plexus and placed on cold EDTA-containing tubes. For the separation of sera, the samples were centrifuged at 2300 ×g and stored at −80°C until required. We used sera for the assessment of blood urea nitrogen (BUN) and serum creatinine (sCr) to evaluate renal function [2]. These parameters were determined spectrophotometrically using commercial kits (Pars Azmoon, Tehran, Iran) as prescribed in the manufacturer's instructions provided with commercial kits.

### 2.7. Kidney Histological Examination

After the animals were euthanized, the right kidneys were isolated and washed with cold saline (0.9% NaCl). After fixation in paraformaldehyde solution (10%) for 24 h at RT, renal parts were dehydrated using ethanol. Paraffin-saturated tissues were preferred for further evaluation, and after 4 h, the samples were fixed on microtome and cut in sections of 4 *µ*m thickness. Then, tissue slides were prepared and stained with hematoxylin-eosin (HE) [17]. Finally, two experienced morphologists randomly examined glomeruli; they were blinded to the origin of the slides.

### 2.8. Evaluation of Oxidative Stress Parameters in Kidney Tissue

#### 2.8.1. Assessment of Glutathione Concentration in Kidney Tissue

An adequate glutathione (GSH) content is necessary in order to maintain the normal function of the kidneys. GSH is measured using DTNB (5, 5′-dithiobis-2-nitrobenzoic acid) as the indicator. Briefly, phosphate buffers and 1 ml of the tissue homogenate were added to trichloroacetic acid. This mixture was centrifuged 1000 ×g for 20 minutes; 1 ml of supernatant was taken and added to 0.4% DTNB and Na_2_PO_4_ and incubated for 15 minutes to complete the reaction. We read the resulting yellow color at 412 nm using a UV spectrophotometer. Glutathione concentration was obtained as µmol/mL (UV-1601 PC, Shimadzu, Japan) based on a calibration curve [18].

#### 2.8.2. Measurement of Lipid Peroxidation Level in Kidney Tissue

Lipid peroxidation (LPO) was determined via quantifying malondialdehyde (MDA) concentrations, which were spectrophotometrically measured via the absorbance of the thiobarbituric acid (TBA) reaction product. In summary, phosphoric acid (0.05 M) was added to tissue homogenate with the addition of 0.3 ml thiobarbituric acid (0.2%). When all tissue homogenates were prepared and previous steps were carried out, they were placed in a water bath (100°C) for 30 minutes. Finally, the samples were taken to an ice bath, with the addition of n-butanol to them. Thereafter, they were centrifuged at 3500 ×g for 10 minutes. Finally, the MDA content was assessed by measuring the absorbance at 532 nm with Epoch™ ELISA reader (Epoch™ Microplate Spectrophotometer, BioteK, USA) according to the calibration curve [19].

### 2.9. Assessment of Mitochondrial Dysfunction

#### 2.9.1. Mitochondrial Preparation

Renal isolated mitochondria were obtained by differential centrifugation technique. The kidneys were homogenized with a glass homogenizer. Essentially, the homogenates were centrifuged for 10 min (at 4°C, 2000 ×g). Then, the centrifugation of the supernatant was performed twice at 10,000 ×g (10 min). Residual mitochondrial pellets were washed and resuspended in cold tris buffer (pH 7). The mitochondrial preparation was performed on ice. Mitochondrial suspensions were used for the assessment of mitochondrial function and oxidative damage parameters [20].

#### 2.9.2. Evaluation of Mitochondrial Function by Complex II Activity Assay

To investigate the mitochondrial function, the activity of succinate dehydrogenase (complex II) was assessed by the 3- [4, 5-dimethylthiazol-2-yl]-2, 5- diphenyltetrazoliumbromide (MTT) assay. Mitochondrial succinate dehydrogenase reduces MTT and produces formazan as a chromophoric product. After the isolation of mitochondria from the kidneys of mice, MTT (0.4%) was added to the isolated mitochondria and incubated at 37°C (30 minutes). The produced formazan was dissolved in dimethyl sulfoxide (DMSO), and the absorbance was measured with Epoch™ Microplate Spectrophotometer (BioteK, USA) at 570 nm [21].

#### 2.9.3. Measurement of Glutathione Concentration in Isolated Renal Mitochondria

As previously explained, glutathione levels were evaluated by using DTNB as the indicator by spectrophotometry. Afterwards, 0.1 ml of isolated renal mitochondria was added to phosphate buffers and DTNB (0.04%) (pH 7.4). The yellow color was read at 412 nm on a UV spectrophotometer (Lambda™ 25 UV-Vis, PerkinElmer, USA) [20].

#### 2.9.4. Measurement of Lipid Peroxidation in Isolated Renal Mitochondria

Malondialdehyde (MDA) was measured based on the method used by Zamani et al. for the evaluation of lipid peroxidation [20]. Briefly, 0.25 ml sulfuric acid (0.05 M) was added to isolated renal mitochondria. In addition, 0.3 ml TBA (0.2%) was added. The tubes were kept in a water bath (100°C) for 30 minutes. Lastly, the tubes were transferred to an ice bath, and 0.4 ml n-butanol was added. Then, the samples were centrifuged for approximately 10 minutes at 3500 ×g. The supernatant absorbance was measured at 532 nm with Epoch™ ELISA reader (Epoch™ Microplate Spectrophotometer, BioteK, USA).

### 2.10. Measurement of Total Protein Concentration

Protein concentration was assessed in tissue homogenates by the Bradford technique. The samples were mixed with Coomassie blue; 10 minutes later, absorbance was determined at 595 nm (Lambda™ 25 UV-Vis, PerkinElmer, USA) [22].

### 2.11. Statistical Analysis

All statistical analyses were performed using GraphPad Prism software, version 6. The results were expressed as mean ± standard deviation. The assays were performed in at least triplicate, and the mean was used for statistical analysis. Comparison between groups was made using the one-way ANOVA test, followed by the *post hoc* Tukey's test. *P* < 0.05 was considered statistically significant.

## 3. Results

### 3.1. Fasting Blood Glucose

**Figure 1:**
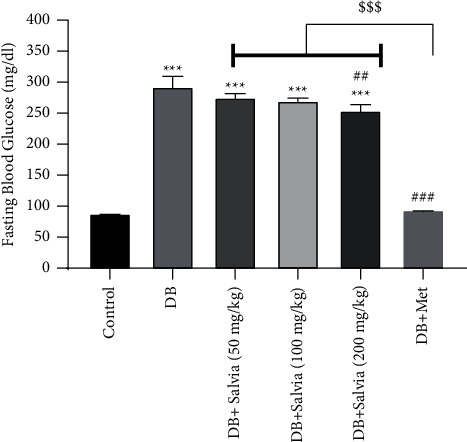
Effect of *Salvia spinosa* extracts on fasting blood glucose concentrations in streptozotocin-induced diabetic mice. Control: normal mice, DB: diabetic mice, DB + Salvia: diabetic mice given the methanolic extract of *Salvia spinosa*, and DB + Met: diabetic mice given metformin (500 mg/kg). Values represented as mean ± SD.  ^*∗∗∗*^*P* < 0.001 compared with control mice, ^##^*P* < 0.01 compared with diabetic mice. ^###^*P* < 0.001 compared with diabetic mice. ^$$$^*P* < 0.05 compared with metformin-treated diabetic mice.

Fasting blood glucose (FBG) is shown in Figure 1. As expected, the untreated diabetic mice had 292.7 ± 19.66 mg/dl, that is, about threefold higher than the control group (*P* < 0.001). Metformin dramatically decreased FBG compared with diabetic mice (*P* < 0.001). *S. spinosa* at 200 mg/kg significantly attenuated FBG compared with diabetic mice (*P* < 0.01); but it was not as effective as metformin-treated diabetic mice (Figure 1).

### 3.2. Kidney/Body Weight Coefficient

**Figure 2:**
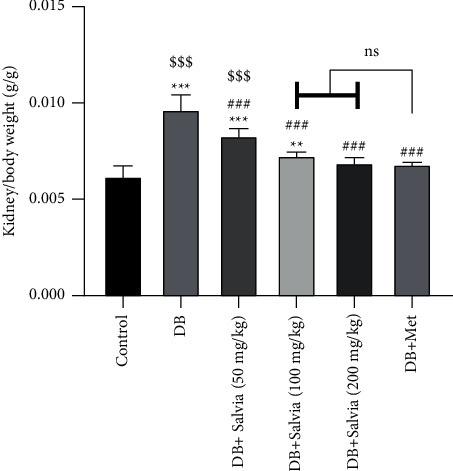
Effect of *Salvia spinosa* extracts on kidney/body weight coefficient in streptozotocin-induced diabetic mice. Control: normal mice, DB: diabetic mice, DB + Salvia: diabetic mice given the methanolic extract of *Salvia spinosa*, and DB + Met: diabetic mice given metformin (500 mg/kg). Values represented as mean ± SD.  ^*∗∗*^*P* < 0.01 compared with control mice,  ^*∗∗∗*^*P* < 0.001 compared with control mice, ^###^*P* < 0.001 compared with diabetic mice. ^$$$^*P* < 0.05 compared with metformin-treated diabetic mice. Ns: nonsignificant.

After 4-week treatment, we measured kidney weight/body weight in the groups. The untreated diabetic mice exhibited increased kidney/body weight (*P* < 0.001 in comparison with control). On the other hand, treated diabetic mice with *S. spinosa* extract and metformin showed a significant weight loss compared to diabetic mice (*P* < 0.001). There were no significant differences in metformin and *S. spinosa* (at 100 and 200 mg/kg)-treated diabetic mice groups (Figure 2).

### 3.3. Biochemical Parameters

**Figure 3:**
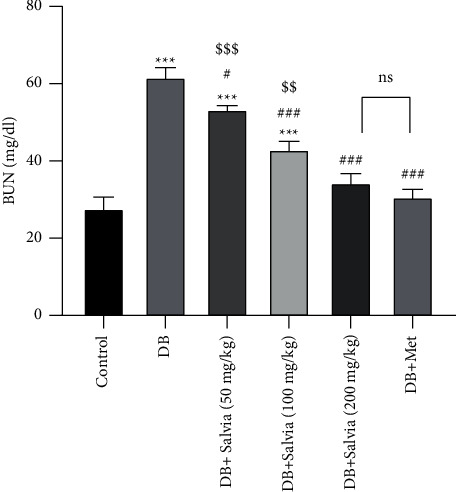
Effect of *Salvia spinosa* extracts on blood urea nitrogen (BUN) concentrations in streptozotocin-induced diabetic mice. Control: normal mice, DB: diabetic mice, DB + Salvia: diabetic mice given the methanolic extract of *Salvia spinosa*, and DB + Met: diabetic mice given metformin (500 mg/kg). Values represented as mean ± SD.  ^*∗∗∗*^*P* < 0.001 compared with control mice. ^#^*P* < 0.05 compared with diabetic mice. ^##^*P* < 0.01 compared with diabetic mice. ^###^*P* < 0.001 compared with diabetic mice. ^$$^*P* < 0.01 compared with metformin-treated diabetic mice. ^$$$^*P* < 0.05 compared with metformin-treated diabetic mice. Ns: nonsignificant.

As shown in Figure 3, the untreated diabetic mice showed increased BUN (*P* < 0.001) compared to control mice. *S. spinosa* and metformin decreased BUN concentrations remarkably compared to diabetic mice (*P* < 0.05). Interestingly, there are no significant changes in BUN concentrations in diabetic mice given *S. spinosa* (at 200 mg/kg) compared to the metformin group (*P* < 0.01) (Figure 3).

**Figure 4:**
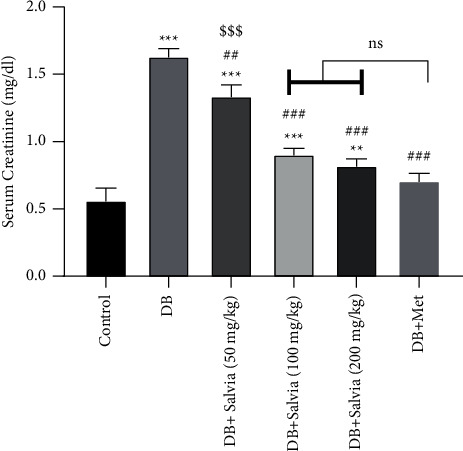
Effect of *Salvia spinosa* extracts on serum creatinine (sCr) concentrations in streptozotocin-induced diabetic mice. Control: normal mice, DB: diabetic mice, DB + Salvia: diabetic mice given the methanolic extract of *Salvia spinosa*, and DB + Met: diabetic mice given metformin (500 mg/kg). Values represented as mean ± SD.  ^*∗∗*^*P* < 0.01 compared with control mice.  ^*∗∗∗*^*P* < 0.001 compared with control mice. ^##^*P* < 0.01 compared with diabetic mice. ^###^*P* < 0.001 compared with diabetic mice. ^$$$^*P* < 0.05 compared with metformin-treated diabetic mice. Ns: nonsignificant.

Furthermore, serum creatinine (sCr), as a marker of GFR estimation, increased significantly in diabetic mice as compared to control mice (*P* < 0.01). The *S. spinosa* treatment considerably reduced sCr in diabetic mice so that the high doses of *S. spinosa* (100 and 200 mg/kg) attenuated sCr in diabetic mice, almost as well as metformin-treated mice. Then, these data demonstrated that *S. spinosa* had a protective effect on kidney function in diabetic mice (Figure 4).

### 3.4. Effect of *S. spinosa* on Histopathology of the Kidney

**Figure 5:**
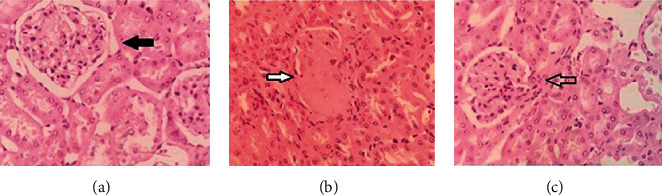
Hematoxylin- and eosin-stained sections of the kidney tissue (×400). (a) Kidney from normal mice showing normal glomeruli (black arrow), (b) kidney from diabetic mice showing the structural disorganization in glomeruli (white arrow), and (c) kidney from *S. spinosa* (200 mg/kg)-treated diabetic mice showing almost normal morphology.

Normal structures of glomeruli and renal tubular epithelial cells were clearly manifested in the kidney fractions of normal control mice (Figure 5(a)). The renal tissue of diabetic mice (Figure 5(b)) showed considerable glomerular and tubular damages represented by tubular necrosis with the vacuolar degeneration of proximal tubules, mononuclear cell infiltration, and thickened basement membranes. *S. spinosa* treatment for 4 weeks significantly ameliorated the severity of the degenerative changes in the renal tissue. Moreover, the mean glomerular volume of the normal control mice was significantly less than that of diabetic mice. The extract considerably reduced the mesangial expansion and glomerular volume compared to the diabetic mice (Figure 5(c)). Thus, our results indicated that *S. spinosa* extract alleviated mesangial expansion and glomerular hypertrophy.

### 3.5. Oxidative Stress Damage in Kidney Tissue

#### 3.5.1. Glutathione Concentration in Kidney Tissue

**Figure 6:**
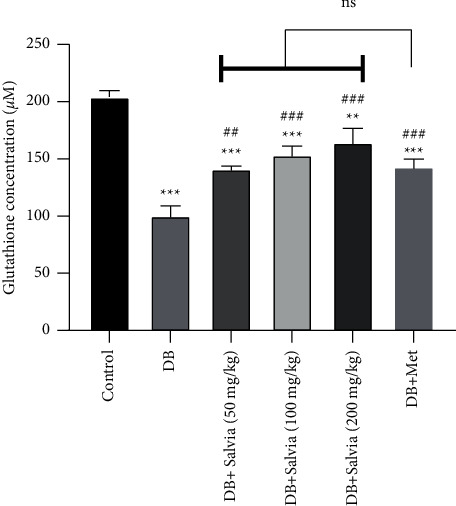
Effect of *Salvia spinosa* extracts on glutathione concentrations in the kidney tissue in streptozotocin-induced diabetic mice. Control: normal mice, DB: diabetic mice, DB + Salvia: diabetic mice given the methanolic extract of *Salvia spinosa*, and DB + Met: diabetic mice given metformin (500 mg/kg). Values represented as mean ± SD.  ^*∗∗*^*P* < 0.01 compared with control mice.  ^*∗∗∗*^*P* < 0.001 compared with control mice. ^###^*P* < 0.001 compared with diabetic mice. Ns: nonsignificant.

Glutathione (GSH) is a main intrinsic antioxidant in tissues, and a decrease in its levels indicates oxidative damage.  Figure 6 demonstrates that GSH concentrations in untreated diabetic mice were approximately half those of normal mice (*P* < 0.001). Treatment with *S. spinosa* at all concentrations increased this content significantly compared with diabetic mice (*P* < 0.001). Interestingly, there were no noteworthy differences between *S. spinosa* and metformin-treated mice (Figure 6).

#### 3.5.2. Lipid Peroxidation in Kidney Tissue

**Figure 7:**
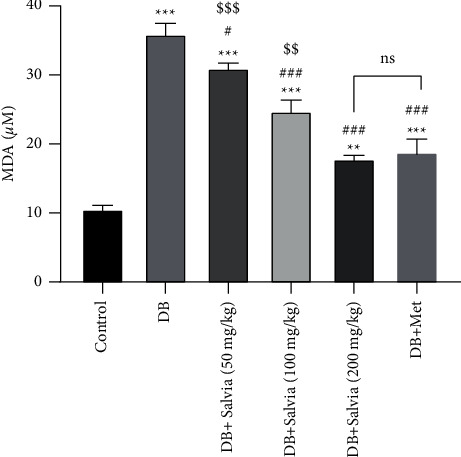
Effect of *Salvia spinosa* extracts on lipid peroxidation in the kidney tissue in streptozotocin-induced diabetic mice. Control: normal mice, DB: diabetic mice, DB + Salvia: diabetic mice given methanolic extract of *Salvia spinosa*, and DB + Met: diabetic mice given metformin (500 mg/kg). Values represented as mean ± SD. ^*∗∗∗*^*P* < 0.001 compared with control mice. ^#^*P* < 0.05 compared with diabetic mice. ^##^*P* < 0.01 compared with diabetic mice. ^###^*P* < 0.001 compared with diabetic mice. ^$$^*P* < 0.01 compared with metformin-treated diabetic mice. ^$$$^*P* < 0.05 compared with metformin-treated diabetic mice. Ns: nonsignificant.

Elevation of MDA (product of lipid peroxidation) is a main marker for oxidative stress. Similarly, in the current experience, diabetes led to an increase in MDA levels from 10.55 ± 0.88 *μ*M in control group mice to 35.90 ± 1.85 *μ*M in untreated diabetic mice (almost fourfold) (*P* < 0.001). Four-week treatment with *S. spinosa* significantly inhibited lipid peroxidation damage compared to the diabetic untreated group (*P* < 0.001). Intriguingly, significant changes were not seen between diabetic mice treated with *S. spinosa* (200 mg/kg) and metformin-treated mice (Figure 7).

### 3.6. Mitochondrial Dysfunction

#### 3.6.1. Effect of *S. spinosa* on Mitochondrial Function (Complex II Activity)

**Figure 8:**
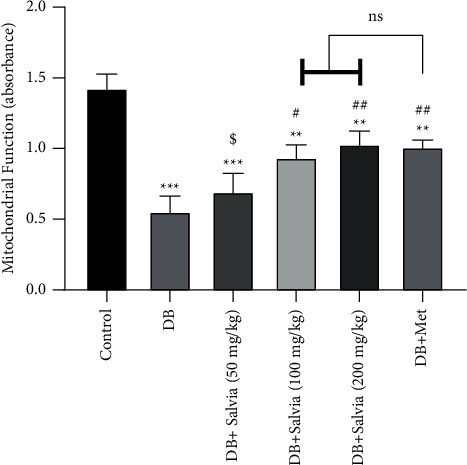
Effect of *Salvia spinosa* extracts on mitochondrial function in renal isolated mitochondria in streptozotocin-induced diabetic mice. Control: normal mice, DB: diabetic mice, DB + Salvia: diabetic mice given the methanolic extract of *Salvia spinosa*, and DB + Met: diabetic mice given metformin (500 mg/kg). Values represented as mean ± SD.  ^*∗∗*^*P* < 0.01 compared with control mice.  ^*∗∗∗*^*P* < 0.001 compared with control mice. ^#^*P* < 0.05 compared with diabetic mice. ^##^*P* < 0.01 compared with diabetic mice. ^$^*P* < 0.05 compared with metformin-treated diabetic mice. Ns: nonsignificant.

We also investigated *S. spinosa* effects on the activity of mitochondrial complex II (succinate dehydrogenase) via the MTT test. After the isolation of renal mitochondria, a significant reduction was observed in the mitochondrial metabolism of MTT to formazan in diabetic mice, compared with normal control mice (*P* < 0.001). *S. spinosa* clearly improved mitochondrial function in diabetic mice (*P* < 0.05). No significant difference was observed in *S. spinosa*-treated mice (100 and 200 mg/kg) in comparison with metformin-treated mice (Figure 8).

#### 3.6.2. Effect of *S. spinosa* on Glutathione Concentration of Isolated Renal Mitochondria

**Figure 9:**
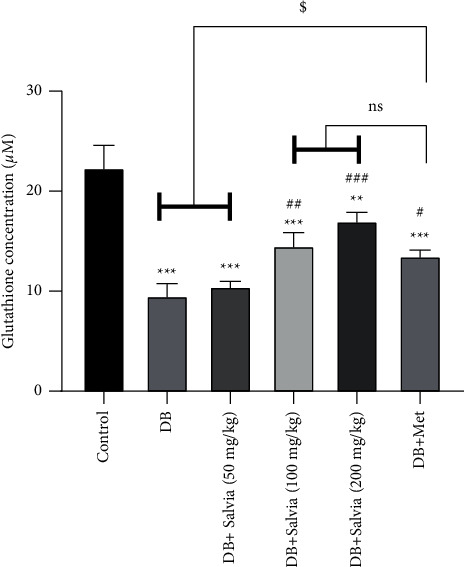
Effect of *Salvia spinosa* extracts on mitochondrial glutathione in renal isolated mitochondria in streptozotocin-induced diabetic mice. Control: normal mice, DB: diabetic mice, DB + Salvia: diabetic mice given the methanolic extract of *Salvia spinosa*, and DB + Met: diabetic mice given metformin (500 mg/kg). Values represented as mean ± SD. ^*∗∗*^*P* < 0.01 compared with control mice. ^*∗∗∗*^*P* < 0.001 compared with control mice. ^#^*P* < 0.05 compared with diabetic mice. ^##^*P* < 0.01 compared with diabetic mice. ^###^*P* < 0.001 compared with diabetic mice. ^$^*P* < 0.05 compared with metformin-treated diabetic mice. Ns: nonsignificant.

The GSH concentrations of diabetic mice were approximately 50% of control groups. As shown in Figure 9, the GSH levels were markedly elevated after *S. spinosa* treatment (100 and 200 mg/kg) (*P* < 0.05 compared with diabetic mice). According to our results, *S. spinosa* represented a protective effect on mitochondrial glutathione contents, specially at 100 and 200 mg/kg doses. Furthermore, the glutathione concentrations of metformin-treated mice were significantly higher than those of diabetic mice (*P* < 0.05). (Figure 9).

#### 3.6.3. Effect of *S. spinosa* on Mitochondrial Lipid Peroxidation

**Figure 10:**
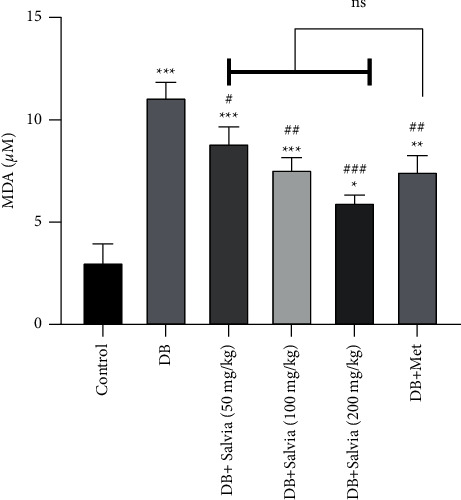
Effect of *Salvia spinosa* extracts on mitochondrial lipid peroxidation in renal isolated mitochondria in streptozotocin-induced diabetic mice. Control: normal mice, DB: diabetic mice, DB + Salvia: diabetic mice given the methanolic extract of *Salvia spinosa*, and DB + Met: diabetic mice given metformin (500 mg/kg). Values represented as mean ± SD. ^*∗*^*P* < 0.05 compared with control mice.  ^*∗∗*^*P* < 0.001 compared with control mice.  ^*∗∗∗*^*P* < 0.001 compared with control mice. ^#^*P* < 0.05 compared with diabetic mice. ^##^*P* < 0.01 compared with diabetic mice. ^###^*P* < 0.001 compared with diabetic mice. Ns: nonsignificant.

As shown in Figure 10, MDA concentration was significantly increased in diabetic mice compared with normal mice (*P* < 0.05). Interestingly, *S. spinosa*-treated diabetic mice (at all doses) significantly decreased mitochondrial lipid peroxidation in diabetic mice (*P* < 0.05 compared with untreated diabetic mice). The significant differences were not shown between *S. spinosa-* and metformin-treated groups (Figure 10).

## 4. Discussion

Diabetic nephropathy is considered the second most prevalent and severe complication of DM. Hypoglycemic, antihypertensive, and lipid-lowering drugs are the conventional medicines prescribed for DM and DN, and these drugs are not totally satisfactory [2, 3, 6, 9]. So, there is the necessity to look for new strategies and treatment for DN's complications. In this study, our focus was essentially on agents with better efficacy and minimum adverse effects.

Dietary and medicinal plants have shown incredible salutary effects on diabetic complications owing to their antioxidants. Herbal antioxidants can activate reduction-oxidation-sensitive transcription factors, and cellular antioxidant and cellular detoxification capabilities [10]. For example, the *Croton hookeri* extract dramatically reduced oxidative stress and renal histopathological damage due to its antioxidant capacity in diabetic rats [5]. As expected, in our study, the diabetic mice showed a rise in lipid peroxidation and GSH oxidation level in the kidney and isolated renal mitochondria, compared with the control group. Four-week treatment with *S. spinosa* led to a significant improvement in DN. Similarly in recent years, Xiang et al. have found that *S. miltiorrhiza* has a therapeutic effect on several complications associated with DM, including DN due to antioxidant properties [3]. Numerous studies have shown that *Salvia* family plants contain numerous flavonoids such as 6, 8-di-C-glucosyl apigenin, apigenin 7-glucoside, luteolin 7-glucoside, luteolin 7-diglucoside, and phenolic acids. Available scientific data currently support beneficial effects of flavonoids and polyphenolic natural products on DN [4, 23]. Flavonoids have been shown to possess phenomenal health-promoting effects. They demonstrated antioxidative and anti-inflammatory effects, and their capacity to modulate key cellular enzyme functions that are associated with antidiabetic properties [4, 10, 24].

Renal hemodynamic changes, lipid disorders, polyol activation, oxidative stress, and inflammatory pathways are known to be several pathogeneses involved in diabetic nephropathy [3, 9, 25]. Moreover, one of the main causes of diabetic nephropathy is oxidative damage caused by persistent hyperglycemia, which can lead to renal mitochondrial dysfunction [10]. In our study, typical signs of DN, such as rise in serum creatinine, BUN, and glomerular hypertrophy, were observed in diabetic mice, and *S. spinosa* treatment provided renal protection by decreasing sCr and BUN as well as by providing antioxidant effects.

The renal cells have a high susceptibility to the hyperglycemia caused by diabetes. Although the complex mechanisms underlying mitochondrial dysfunction in diabetic nephropathy are not fully assumed [1], hyperglycemia leads to an increase in intracellular glucose and acceleration of mitochondrial oxidative phosphorylation with the excessive leakage of single electrons to the oxygen molecule (O_2_), thereby forming superoxide (O_2_^−^), leading to other reactive oxygen species (ROS). Minimal levels of ROS are detoxified by cellular antioxidants such as glutathione, catalase, and superoxide dismutase [26]. Hypothetically, excessive oxidative stress damages mitochondria, proteins, and membranes, resulting in mitoptosis (localized mitochondrial destruction). As demonstrated in our results, a significant plummet in mitochondrial function was observed in diabetic mice compared to the control group, and we have indicated that *S. spinosa* led to a remarkable amelioration of mitochondrial toxicity [10]. In addition, lipid peroxidation decreased, and GSH amounts normalized (in kidneys and isolated renal mitochondria) in 4 weeks of treatment. Similarly, other studies demonstrated that the antioxidants such as coenzyme Q10 had a nephroprotective effect in DN by preventing mitochondrial oxidative damage. Therefore, as shown in results, *S. spinosa* reduced the level of oxidative stress and led to mitochondrial protecting effects in the presence of diabetes-induced mitochondrial damage.

In addition, the ROS along with oxidized proteins, lipids, nucleic acids, and carbohydrates contribute to cellular dysfunction and compromised integrity, and eventually to apoptosis (programmed cell death) [26]. These lead to such histopathological changes as glomerular hypertrophy and promote fibrogenesis in the glomeruli and tubules [25]. Furthermore, our results of kidney histopathology revealed the alleviated glomerular hypertrophy and mesangial expansion in diabetic mice, showing that these damages were improved after *S. spinosa* treatment. Similar to our results, Trujillo et al. showed that antioxidant plants such as turmeric had nephron-protective effects due to the inhibition of mitochondrial dysfunction and prevention of oxidative stress [27], as well as preventing lipid peroxidation [28, 29]. Thus, *S. spinosa* can probably alleviate diabetic nephrotoxicity via protecting mitochondria, by reducing oxidative stress. *S. spinosa* combined with antihyperglycemic agents could be highly effective in the treatment of diabetic nephropathy. Nevertheless, the lack of phytochemical analysis is a limitation of our study. As previous data suggest, the quality of plant materials is associated with the geographic and ecological conditions and phytochemical analysis should be carried out in subsequent studies.

## 5. Conclusions

In summary, advanced oxidative stress is a probable mechanism, which contributes to the diabetic complications, including nephropathy. To the best of our knowledge, our study has shown for the first time that *Salvia spinosa* effectively decreased oxidative stress and renal injury in STZ-induced diabetic mice. *S. spinosa* reduced the levels of sCr and BUN and improved the histopathological state in the diabetic mice. Moreover, *S. spinosa* significantly prevented mitochondrial dysfunction. The results suggest that in the treatment of diabetic nephropathy, *S. spinosa* is a potential therapeutic agent. Therefore, it is necessary to carry out further exploration for authentic data to justify clinical application of this plant against diabetic complications, besides other blood glucose-lowering protocols.

## Data Availability

Data of this study are available from the corresponding author upon reasonable request.
